# Supplementary material for: Monolayer Capping Provides Close to Optimal Resistance to Laser Dewetting of Au Films
Source: ACS Appl Electron Mater. 2023 Aug 4;5(8):4080–93. doi: 10.1021/acsaelm.3c00052 (PMC10448724; doi:10.1021/acsaelm.3c00052)
Supplement: Supplementary file 1 — el3c00052_si_001.pdf [file el3c00052_si_001.pdf]

Supporting Information for

Monolayer capping provides close to optimal resistance to laser dewetting of Au films

Christopher P. Murray[1], Daniyar Mamyraimov[1], Mugahid Ali[1], Clive Downing[1], Ian M. Povey[2], David McCloskey[1], David D. O'Regan[1] and John F. Donegan[1]*

[1]School of Physics, CRANN and AMBER, Trinity College Dublin, The University of Dublin, Dublin 2, Ireland.

[2]Tyndall National Institute, Lee Maltings, Prospect Row, Cork, Ireland

Corresponding Author

Christopher P. Murray,

School of Physics, CRANN, & AMBER,

Trinity College Dublin,

Dublin 2,

Ireland

https://orcid.org/0000-0002-7496-9442

Email: murrayc2@tcd.ie

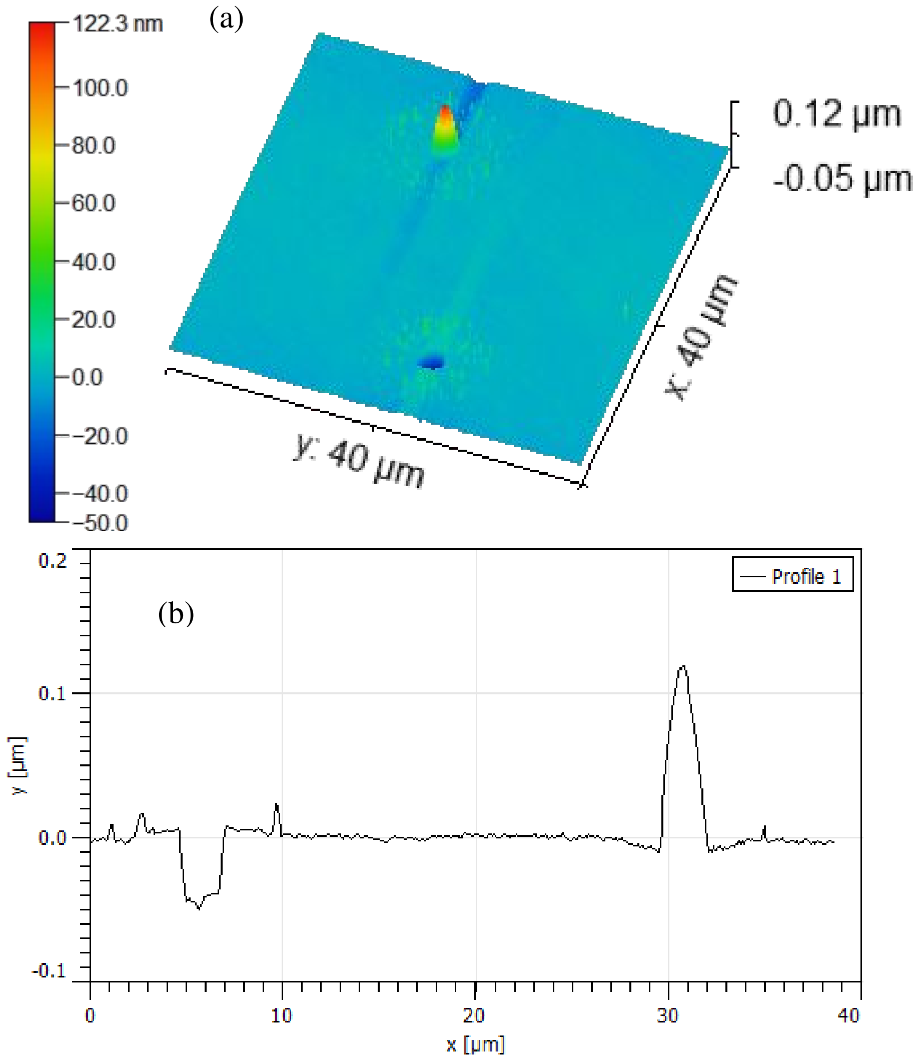

Figure S1. AlOx 5 nm capped sample post dewetting at P_{abs} = 40 mW and (b) a cross section through the centre of both dewet areas. While one retains a cap which bulges upwards by 130 nm, the other has broken through revealing a hole which extends down to the substrate.

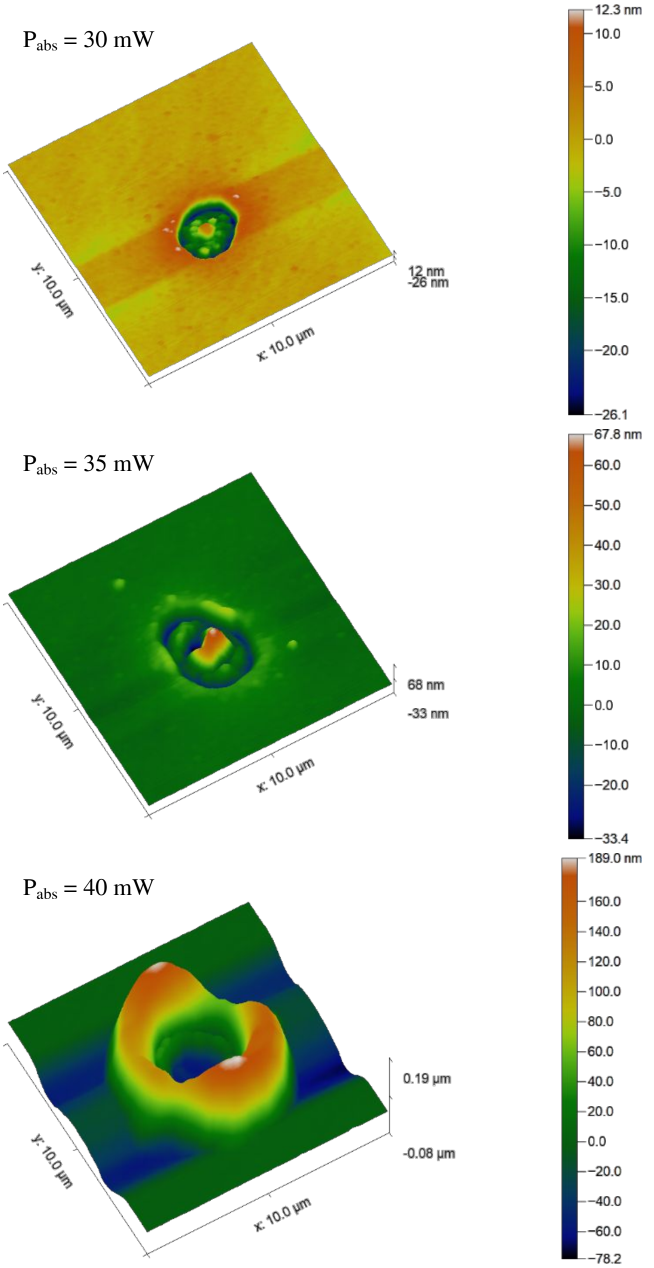

Figure S2. AFM topography of Ta 0.5 nm / Au 50 nm / Al 5 nm capped sample post dewetting at P_{abs} = 30, 35 and 40 mW

For the Al 5 nm capped samples, no remaining capping material covers the dewet area (Figure S2). Instead, dewet material either remains in the center of the area (P_{abs} = 30 and 35 mW) or piles up at the edges of the area (P_{abs} = 40 mW) leaving a crater-like feature. As Al forms a self-limiting oxide of c.4 nm, this sample is the only one which has metal rather than oxide in contact with the Au, and this is likely responsible for its unique response – it is the worst performing sample, worse even than having no capping layer at all. In contrast, for capping layers of 0.5 nm, no remnant capping layer was found covering the dewet area (Figure S4). The boundary of the dewet area is quite rough, and some Au remains in the dewet area.

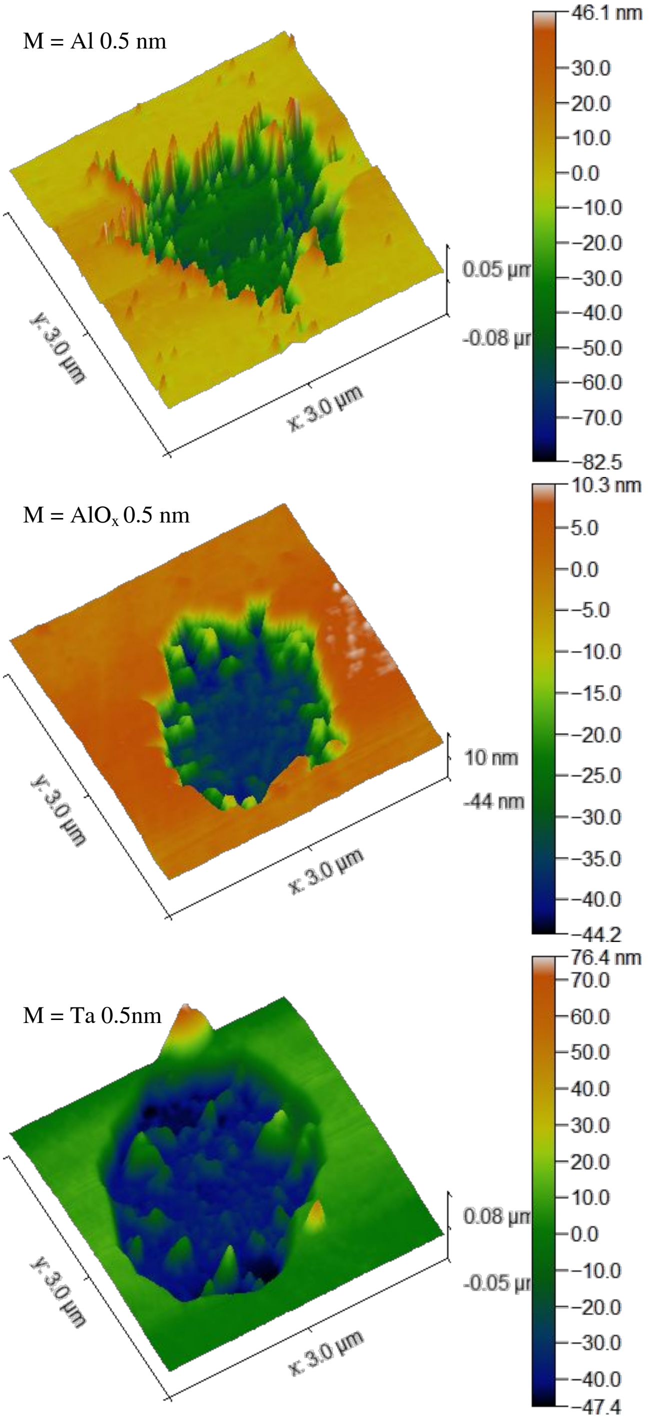

Figure S3. AFM topography of the Ta 0.5 nm / Au 50 nm / M 0.5 nm samples post dewetting at P_{abs} = 40 mW
